# "The Hospital" Medical Book Supplement.—No. XXVI

**Published:** 1910-02-19

**Authors:** 


### "THE HOSPITAL"

### MEDICAL BOOK SUPPLEMENT.—No. XXVI.

CONTENTS.

| | Page |
|---|---|
| **Medicine—** | |
| Essentials of Medical Electricity and Radiography ... ... | 601 |
| The Medico-Chirurgical Series, No. 2: the Stomach, Intestines, and Pancreas ... ... ... ... ... ... ... | 601 |
| **Surgery—** | |
| Lectures on Surgical Nursing ... ... ... ... ... ... | 602 |
| The Practice of Surgery ... ... ... ... ... ... ... | 602 |
| Male Diseases in General Practice ... ... ... ... ... | 603 |
| **Medico-Legal—** | |
| The Law Relating to Poisons and Pharmacy, with Notes on Cases ... ... ... ... ... ... ... ... ... | 604 |
| **Miscellaneous—** | |
| Willing's Press Guide, 1910 ... ... ... ... ... ... | 604 |
| Essentials of Chemical Physiology ... ... ... ... ... | 604 |
| Religion and Insanity ... ... ... ... ... ... ... | 604 |
| A Beautiful Arm ... ... ... ... ... ... ... ... | 604 |
| The Science Year Book ... ... ... ... ... ... ... | 604 |

### MEDICINE.

ESSENTIALS OF MEDICAL ELECTRICITY AND RADIOGRAPHY. By EDWARD R. MORTON, M.D., F.R.C.S. Ed. Second edition. (London: Henry Kimpton, 13 Furnival Street, E.C. 6s. net.)

WE are glad to welcome the second edition of this useful little handbook, one of Mr. Kimpton's excellent "Essential Series." The section on radiography, which is a new addition, is a clear and concise epitome of the essentials of *x*-ray technique. To some extent the work is dogmatic, but that in a volume of this nature is unavoidable, and indeed necessary. The illustrations are really helpful, while brevity and clarity are everywhere characteristics of the text. The practitioner will find this little manual one of the best reference handbooks on the subject it deals with.

THE MEDICO-CHIRURGICAL SERIES, No. 2: THE STOMACH, INTESTINES, AND PANCREAS. By W. C. BOSANQUET, M.D., F.R.C.P., and H. S. CLOGG, M.S., F.R.C.S. Edited by JAMES CANTLIE, F.R.C.S. (London: John Bale, Sons and Danielsson, Ltd. 1909. Pp. 665.

IT is always of interest in perusing the work of collaborators to endeavour to disentangle the personalities of the authors, and to detect which of them is responsible for which sections. In the case of collaboration between a physician and a surgeon in the authorship of a textbook, this separation is rendered easier by the natural affinities of the subject under discussion; one does not expect to find a physician dealing with intestinal anastomosis, any more than one expects a surgeon to have much to say about ankylostomiasis. But, apart from this, it is not difficult as a rule to recognise the respective handiwork of these two authors. Mr. Clogg has, unless we are mistaken, undertaken by far the larger part of the present volume. His style, though far from academical perfection, is vigorous and even trenchant; and he is a surgeon with convictions and the courage of them. Dr. Bosanquet is less inclined to be dogmatic, and for this reason is perhaps less forceful in effect than his colleague. He has also allowed himself to be somewhat elbowed out, as, for instance, when he claims but just over three pages for the discussion of infantile diarrhœa, whereas Mr. Clogg takes seven and a half for the much less important subject of congenital hypertrophic stenosis of the pylorus. This has resulted in a certain lack of balance between the surgical and medical sections of the book, to the disadvantage of the latter; thus there is no mention of whey as an article of infant diet, an omission no doubt due to the scanty room allotted to the extensive subject of intestinal disorders in infancy.

Apart from this we are able to extend a large measure of praise to this second volume of the Medico-Chirurgical Series. It is well printed in large and clear type, and it is about the right size of a regional textbook for the general practitioner—that is to say, it is not so encyclopædic as to be wearisome, nor so brief as to be unduly elementary. The subjects are treated in a practical and thoroughly up-to-date way, and diagnosis and treatment receive the full share of attention which they deserve. It would be strange if here and there views were not advocated with which some do not agree. Thus Mr. Clogg is opposed to the use of antiseptics in the preparation of the skin of a baby which is to be operated upon for pyloric stenosis. He is also, in our judgment, unnecessarily diffuse about bands and the different ways in which they may strangulate the intestines; congenital atresia of the intestines is another subject dealt with at a length quite inordinate to its importance. Abdomino-perineal excision of the rectum and pelvic colon for malignant disease as now practised is, on the other hand, not described. In speaking of gastric cancer, two common symptoms are not mentioned; one is the curious variability from day to day in the size and position of a pyloric tumour, when it exists; and the other is the afternoon somnolence so often noted in quite early stages of this complaint. Constipation is common, as Dr. Bosanquet remarks, in civilised races; but it is in fact perhaps as common in savages, so that the non-stimulating character of overcooked food is not the main factor at work in promoting constipation. Then it can hardly be correct to say that colic is usually, if not always, associated with constipation; as a matter of experience, surely the severest forms of intestinal colic are quite frequently seen in connection with diarrhœa and vomiting in cases of food poisoning. In the discussion of appendicitis and other inflammatory diseases of the peritoneum no mention is made of the work of Head, Sherren, and others on referred cutaneous tenderness. Apart from these points, none of which is, of course, of vital importance, we can most thoroughly recommend this volume to the profession, whose members will find the association of a physician and surgeon fraught with very great benefit to the readers of this very able and satisfactory monograph.

### SURGERY.

Lectures on Surgical Nursing. By E. Stanmore Bishop, F.R.C.S. (Bristol : John Wright and Sons, Ltd. 1909. Price, leather, 3s. 6d.; cloth, 2s. 6d.)

In this volume are printed a series of twelve lectures delivered by the author to nurses at Manchester. It is with somewhat mixed feelings that a medical man peruses them, for there is much in them that throws light on the shortcomings of the medical profession. That is to say, there is instruction given on matters which are (or should be) completely outside the nurse's province; yet evidently medical men, in Lancashire at least, are in the habit of delegating some of their most important duties to nurses. For the opening chapter on the history of antisepsis and asepsis we have nothing but praise; to describe the pre-Listerian era and to explain the *rationale* of the technique which has abolished its horrors, is to give nurses an insight into first principles which must help them to understand what they are taught. Sterilisation, chart-taking, baths, theatre duties, and other important subjects are also adequately dealt with. It is in the seventh and twelfth lectures that Mr. Bishop endeavours to lead the nurse outside her province. In the former he goes into details of urine testing, examination of the fæces, and even gastric lavage and examination of its products, in a way which requires an emphatic protest. It is not desirable, in our opinion, that these delicate and difficult analyses—many of which practitioners and surgeons themselves delegate entirely to pathological experts—should be entrusted to nurses, and it is not fair to them to expect them without any preliminary scientific training to grasp the principles and practice involved. In the latter he describes the catheterisation of the male urethra by silver instruments as a duty nurses may be called upon to undertake; the use of soft catheters is said to be too easy to require description! We can only say that these pages are a very grave indictment of the medical profession.

The Practice of Surgery. By W. G. Spencer, M.S., F.R.C.S., and G. E. Gask, F.R.C.S. (London : J. and A. Churchill, 7 Great Marlborough Street. 1910. 22s. net.)

A very large percentage of practitioners know Walsham and Spencer so well that it is a work of superabundance to dilate on the merits of this popular and eminently serviceable textbook of surgery. Walsham and Spencer now in this the tenth edition becomes Spencer and Gask, and we feel confident that the change of name will in no way impair the popularity of the work, while this edition will do much to enhance its reputation as one of the best manuals of practical surgery that the student or practitioner can buy. The historic association that the work has with the oldest London hospital has been retained through the co-operation of Mr. Gask, who is on the surgical staff at St. Bartholomew's, and who is jointly responsible for the book, while Mr. Jessop, as in former editions, deals with the diseases of the eye, and Mr. West with those of the ear.

We note a variety of improvements in this edition, chief among which is perhaps the excellent colour illustrations. Some of these remind one of the well-known pictures in Hutchinson's "Illustrations of Clinical Surgery," and are not merely accurate delineations in colour of important lesions, but veritable works of art so far as detail is concerned. We regret, however, to see so many micro-photographs used as illustrations. They never reproduce well in print, and some of those in this edition, notably on page 139, where a soft spheroidal-celled carcinoma is depicted, and on page 151, where the illustration is said to be that of a labial obstruction cyst, are execrable. In our opinion it would be much more advantageous to the student to delete these illustrations altogether, and supply in their place diagrammatic or semi-diagrammatic drawings, showing accurately or schematically the condition present on microscopic examination. Such diagrams are already given (Figs. 34, 3, 13, 72, and others), and there can be little doubt that their educative value transcends that of the micro-photographs. The only other way of illustrating the microscopic appearance of sections is by means of accurately drawn and coloured illustrations—the ideal way and one that we should like to see adopted in a future edition. The block illustrations, many of which are new, are all good in so far as they elucidate the text. Some of them, especially the photographs of actual cases and the well-chosen skiagraphs, leave nothing to be desired, but we think one or two more actual photographs might with advantage have been included in order to show clinical signs. Thus in the section on congenital dislocation of the hip a brief account of Trendelenburg's sign (of which no mention is made under sacro-iliac disease or hip joint disease) is given. This is an advance upon most textbooks, which absolutely ignore this useful clinical sign; but at the same time the value of the description from a student's point of view would have been increased by the addition of a photograph showing the dropping of the pelvis and the manner of eliciting the sign.

The arrangement of the book into sections does not differ from preceding editions. In the section dealing with general pathology we notice the statement that the Widal reaction is "useful sometimes to distinguish between general peritonitis and typhoid," while the opsonic index is curtly dealt with in three lines. It is only far to say that the importance of opsonic treatment is frankly referred to further on. No mention is made of sporotrichosis, which we should have thought is by this time sufficiently well established as a clinical entity to deserve a place in a textbook. Scalvo's serum and carbolic injections are the only therapeutic agents mentioned in the treatment of anthrax : a practitioner who has no serum available might have liked to hear something about the usefulness of ipecacuanha. We think the authors are right in not countenancing the indiscriminate use of rubber gloves in operating : the careful and methodical cleansing of the hands on which they lay stress is a point that should be insisted upon by all teachers. The chapters on operative procedure, preparation, anæsthetics, wounds, and hæmorrhage are excellent, and practitioners may gain many useful hints by reading them. In the chapter dealing with anæsthesia little is said about recent improvements, while the use of pituitary extract in cases of shock is not mentioned. The chapter on nerve and muscle surgery contains many references to recent work, and is admirably clear and concise, avoiding unnecessary minutiæ, while clearly explaining the better proven methods of nerve grafting and tendon surgery. That on fractures contains little that is strikingly novel, but a vast amount of exceedingly practical clinical instruction which should prove of help to students and practitioners alike. The authors are in line with the best surgical opinion of the day in recommending a simple sling and bandage, after thorough rectification of deformity, as the best treatment in cases of Colles's fracture, and in condemning splinting for this lesion. We doubt if they will be so generally supported in their opinion that forcible reduction of a shoulder dislocation, whether recent or old standing, is obsolete, and in their practice of dealing with all such injuries that have withstood gentle manipulative attempts at reduction, by means of the open method. Nor do we think that practitioners will agree with them in their treatment of fracture of the radius above the pronator by a position midway between pronation and supination. We fully agree that, from a patient's point of view, this is the most pleasant way of fixing the arm, and that such fractures heal well, with excellent ability to pronate and supinate, provided that care is taken in starting passive and active movements early; but we are not convinced that for a general practitioner who cannot give much time to these cases, the old method of splinting in the fully supinated position, however irksome to the patient, is to be preferred in dealing with such fractures. In the sections dealing with diseases and deformities, diseases of bone, and the surgery of the skull and brain, much new matter has been incorporated, and the first is indeed one of the best chapters on orthopædics to be found in any text-book. The diagnosis of intracranial lesion, especially the localising symptoms and signs, are somewhat too briefly treated : a fuller account would be more helpful to practitioners.

We have no space to take in detail the remaining chapters of this work of 1,200 odd pages, but it is sufficient to say that the new material has been carefully written, and the old as carefully revised, so that the book is thoroughly up to date, though of necessity dogmatic and brief in some respects. The surgery of the abdomen in particular has received special attention in this edition, and the sections dealing with the stomach and intestines are very complete. Mention is made of Dalton's method of $x$-ray examination in stomach cases, and of recent opinion on the subject of duodenal ulcer. The section on appendicitis is clear, and is moreover illustrated by a fine coloured plate showing concretions in and inflammation of the appendix : as is to be expected from one of the author's known opinion on the subject of early operation, the case for operative interference in an acute attack is very well put. Hernia, fæcal fistula, and abscess are considered at some length, while the chapters dealing with genito-urinary diseases and diseases of women, with injuries and diseases of the eye and with ear affections, are as clear and as excellent as the rest of the book. The index is fairly good, but can scarcely be called perfect.

In our opinion there is no better textbook of general surgery in the English language available for students at present than this edition of Spencer and Gask. We say so with a full sense of the responsibility of judging between the several good manuals in the market. For students' purposes it is almost ideal, and as it is comparatively cheap and fairly handy in size (though more bulky than its predecessors), it is bound to become increasingly popular. For the general practitioner it is perhaps less useful, because it does not go deeply into questions of treatment; but it is a useful reference manual, sound in teaching, and will therefore prove of help to practitioners who desire to get a thoroughly up-to-date standard work on surgery.

Male Diseases in General Practice. An Introduction to Andrology. By Edred M. Corner, M.C., F.R.C.S. (London : Henry Frowde and Hodder and Stoughton. Oxford Medical Publications. 15s. net.)

In his preface Mr. Corner remarks that "a special plea for the foundation of a science of andrology is that men are without doubt carriers and transmitters, often unconsciously, of venereal infection into their own homes. The lives of many women are spoiled through the unconscious ignorance of the men they marry." We would have thought this state of affairs—of the truth of which every practitioner is fully aware—was to be remedied not by the creation of a separate specialism, but by coping directly with this popular ignorance. In other words, that the time has come for the profession to take the public more fully into its confidence, especially on matters of sex and genito-urinary disease. That andrology, as such, is well worth special study we do not for a moment deny. At several Continental hospitals there is a department specially devoted to it; and though specialism in this country has not progressed so far as yet, it is probably only a matter of time before some at least of our general hospitals will have their andrological departments. We are glad to note that Mr. Corner is not an exclusivist who desires to separate his speciality entirely from general surgery, and that he insists upon the imperative necessity of the future andrologist acquiring a sound general training. These points are worth consideration, for there is a natural tendency to regard "special textbooks" as being the hobby horses of their writers, and the employment of the new term is likely to scare off the average reader.

That, we venture to think, is not a danger in his case, for the book, apart from its somewhat uncouth and absurd title, is wholly good. It is one that can be confidently and frankly recommended to the general practitioner, for it is one that will help him in his diagnosis and treatment of a class of case that he meets with very often. It is, like all the Oxford manuals, well printed and illustrated, and written in a clear, lucid manner which, while it makes no pretence to being literary and at times gives the reader the impression that the writer was in a hurry, is concise and forcible. Its matter is sound, but we regret that Mr. Corner has not held very clearly before him the fact that the book is primarily destined for the practitioner, and that he has taken up space with a discussion of abstract points, or matters of scientific interest, to the detriment of his descriptions of matters of far greater practical importance. It is very interesting to know that in certain savage races the right testicle is lower than the left—it is certainly not so in all—but it is far more important, in practice, to know how to distinguish between a tuberculous and a syphilitic testis. Mr. Corner devotes more than 20 pages to incomplete statistics regarding the relative position of the testis—drawing from them the wholly unproved conclusion that the "lower level of the left testicle found in civilised races is an acquired feature dependent on the wearing of their apparel"—and dismisses the more important question of the differential diagnosis between tubercle and syphilis in as many paragraphs. We should have much preferred to see a more minute account of the clinical differences between the two forms of testicular disease, and we certainly must take exception to the statement that when testis and epididymis are indistinguishable the differential diagnosis is "mere pedantry." On the functional affections, which again from a point of view of practice are important, the author is very clear, and this chapter is one of the best in the book, although here again too much attention has been paid to subjects like hermaphroditism and azoospermia, in which the practitioner will hardly take practical interest. The chapters dealing with the urethra and the prostate contain much excellent clinical teaching, and they are the more praiseworthy since they are so concise and clear. We note with satisfaction the advice given on page 310 with regard to narcotics and the inadvisability of administering bromides in cases of prostatitis. Equally sound and excellent is the teaching in the section dealing with urethral discharges, urethral fistula, and stricture. A feature of the book is the résumé of illustrative cases, which adds much to the value of the various chapters. There are in addition some specially interesting features, especially in the first part. Thus torsion of the testicle is admirably described, and as it is an accident which not infrequently occurs in practice, and is not always easy to diagnose, the chapter dealing with it is well worth reading. Taken as a whole the book is a very good summary of present-day teaching in diagnosis and treatment of the male generative organs, and no practitioner who is interested in this branch of surgery will regret placing it upon his shelves.

### MEDICO-LEGAL.

The Law Relating to Poisons and Pharmacy, with Notes on Cases. By W. S. Glyn-Jones, Barrister-at-law. Pp. xxxvi.+410+21. Price 10s. 6d. (London: Butterworth and Co. 1909.)

All medical practitioners, and especially those who have any responsibility for the management of a public or private dispensary, may acquire much useful knowledge by studying the law relating to the storage and distribution of poisons. It is true that, largely through the watchfulness of the representative bodies of the profession, the Pharmacy and Arsenic Acts contain clauses which safeguard, more or less effectively, the right of medical practitioners to dispense their own medicines as may to them seem fit and in the interests of their patients. But in spite of exemptions, the requirements of the statutes with respect to the handling of poisons should certainly be known and in many cases observed by practitioners of medicine, and in the dispensaries of public institutions, no less carefully than in a pharmacist's shop. That there are lacunæ in the Pharmacy Acts is evident. It will suffice to quote the omission to require any educational test or qualification for persons who purport to dispense medical prescriptions in pharmacists' shops, and the absence of any check on "counter-prescribing." The schedule of poisons, again, is not yet quite satisfactory; but its defects may be repaired by the Pharmaceutical Society with the consent of the Privy Council. Mr. Glyn-Jones has unique qualifications for writing on the subject, and it is understood that but for his work as Parliamentary Secretary of the Pharmaceutical Society, the Poisons and Pharmacy Act of 1908 would not have reached the statute-book. In this book Mr. Glyn-Jones not only deals fully with this Act, but presents a lucid survey of the whole field of legislative restrictions on the sale of poisons and the practice of pharmacy. Incidentally, the provisions of the Apothecaries Act with reference to "practising as an apothecary," and those of the Dentists Act, 1878, with reference to the use of professional titles, are discussed at some length. Coroners and all who have to do with cases of poisoning will find the book invaluable, while medical officers of health and medical men who are members of local authorities will be specially interested in Chapter V., on the powers and duties of local Councils with respect to the granting of licenses for the sale of agricultural and horticultural poisons. To officers of hospitals and similar institutions Chapter II., on the regulations for the keeping of poisons, will specially appeal. There was need for a satisfactory book dealing fully with the law relating to poisons and pharmacy. This need Mr. Glyn-Jones appears to have satisfied thoroughly well.

### MISCELLANEOUS.

Willing's Press Guide, 1910. (James Willing, Jun., 125 Strand.)

This is the thirty-seventh annual issue of a useful reference book. It gives brief details of every publication issued in Great Britain and the Colonies, and of the principal publications of the world. The volume is of a handy size and well printed. A good feature is the list of Metropolitan publications arranged according to the dates of issue.

Essentials of Chemical Physiology. By Professor Halliburton. Seventh Edition, 1909. (Messrs. Longmans, Green and Co. 280 pages. 71 Illustrations. Price 4s. 6d. net.)

This remarkably useful textbook has been thoroughly revised and brought up to date, without its bulk being appreciably increased. We note that a few pages are devoted to the subject of Lipoids, and a section on organic compounds is also wisely incorporated in the new edition.

Religion and Insanity. By Charles Williams, L.R.C.P., L.R.C.S., L.S.A. (The Ambrose Co., Ltd., 55 and 57 Wigmore Street, W.).

These pages set forth the author's belief that one of the principal causes of insanity is loss of faith in the Christian religion. The second part, moreover, describes various religious methods for the treatment of insanity, and an appendix contains the forms or services for the administration of unction, or imposition of hands, as well as several excerpts from the Roman Catholic service for exorcism.

A Beautiful Arm. By Amos Booth. (London: Sampson Low, Marston and Co. 2s. net.)

This volume is directed against vaccination, and from cover to cover is full of the various crazes, delusions, and superstitions that are born in the anti-vaccinationist's brain. One naturally wonders why a book should bear such an extraordinary title; but in the preface the reason is given. In a note the author states that the publication has been directly instigated by a Mr. Taylor of California, whose portrait has been added to enhance the value of the work. It is a comfort to reflect that the production of such rubbish is not wholly due to the distorted views of an English mind.

The Science Year-book. (King, Snell and Olding, Ltd., 27 Chancery Lane, W.C. 1910. 5s. net.)

This excellent Year-book is of real service to a medical or scientific man. It contains much useful information; and as one of the bits of less well known fact included in it we may cite the following details about the Wolfskehl prize: "Dr. Paul Wolfskehl, of Darmstadt, left in his will provision for payment of £5,000 to the first person who would afford the mathematical proof of the Fermat formula for all quantities. This formula is: While the sum of two squares of whole numbers may amount to the square of another whole number, the sum of two cubes can never be the cube of a whole number. The award of the prize rests with the Göttinger Gesellschaft der Wissenschaften." Here is a chance for the mathematically minded doctor in these difficult days when practice is poor! The list of scientific societies given in the book is full but not quite accurate in all respects, especially in the case of Colonial societies.

